# Supplementary material for: Rhizobacteria opportunistically boost colonization and impair plant fitness by degrading plant-derived coumarins under iron deficiency
Source: Nat Commun. 2026 Mar 25;17:4398. doi: 10.1038/s41467-026-71037-3 (PMC13181038; doi:10.1038/s41467-026-71037-3)
Supplement: Supplementary file 1 — Supplementary Information [file 41467_2026_71037_MOESM1_ESM.pdf]

Supplementary Information

Rhizobacteria opportunistically boost colonization and impair plant fitness by degrading plant-derived coumarins under iron deficiency

Yichao Gu[a], Piaopiao Pan[a], Gang Yu[b], Ning-Yi Zhou[a*]

[a]State Key Laboratory of Microbial Metabolism, Joint International Research Laboratory of Metabolic and Developmental Sciences, and School of Life Sciences and Biotechnology, Shanghai Jiao Tong University, Shanghai, China

[b] Shanghai Collaborative Innovation Center of Agri-Seeds, Joint Center for Single Cell Biology, School of Agriculture and Biology, Shanghai Jiao Tong University, Shanghai, 200240, China

*Correspondence to Ning-Yi Zhou, ningyi.zhou@sjtu.edu.cn.

Supplementary figures

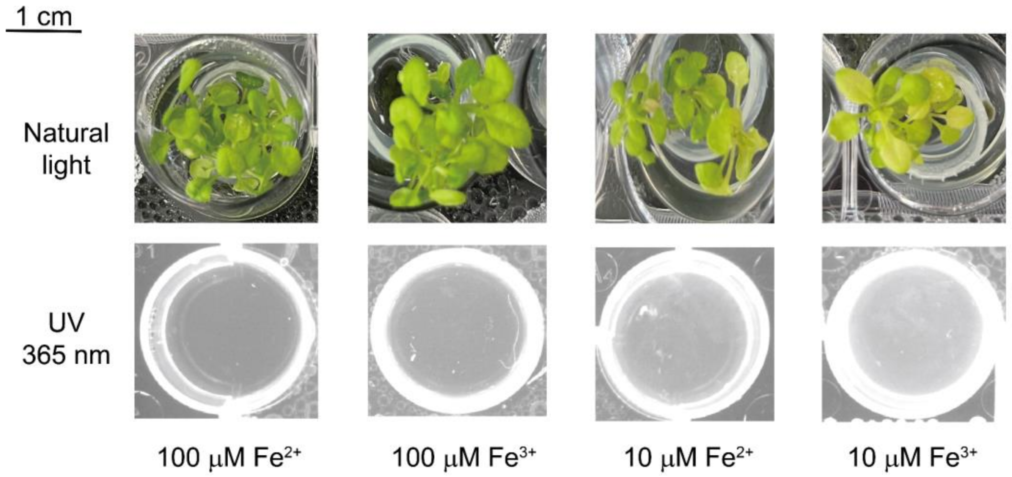

Supplementary Fig. 1: Phenotypic characterization of Arabidopsis Col-0 with varying iron supplementation and preliminary determination of simple coumarin exudation by plants. Representative images of 18-day-old hydroponically grown plants under natural and UV (365 nm) light, showing phenotypic responses to varying iron availability in the liquid ½ Murashige Skoog (½ MS) medium.

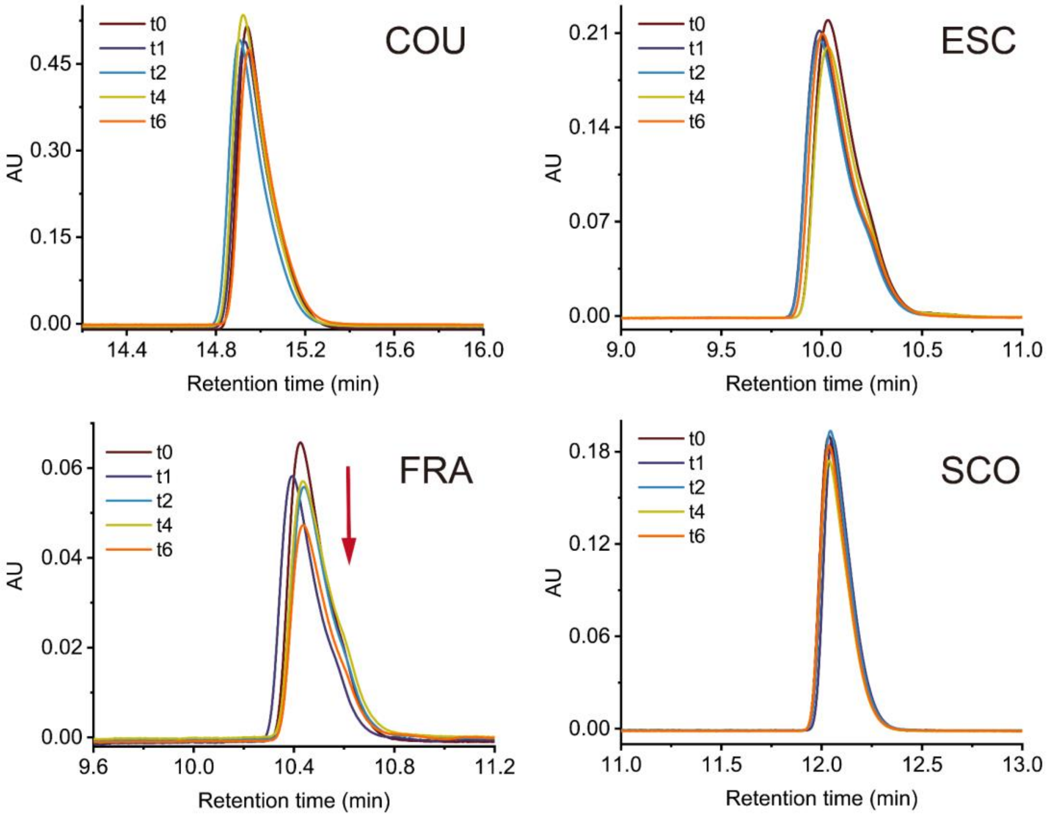

Supplementary Fig. 2: Stability of simple coumarins monitored by HPLC. The stability of coumarin (COU), esculetin (ESC), fraxetin (FRA), and scopoletin (SCO) was tested in minimal medium (MM), with each compound supplied at 0.5 mM. The concentrations were monitored over 6 hours (0, 1, 2, 4, 6 h) by HPLC. The red arrow indicates the degradation of compounds over time.

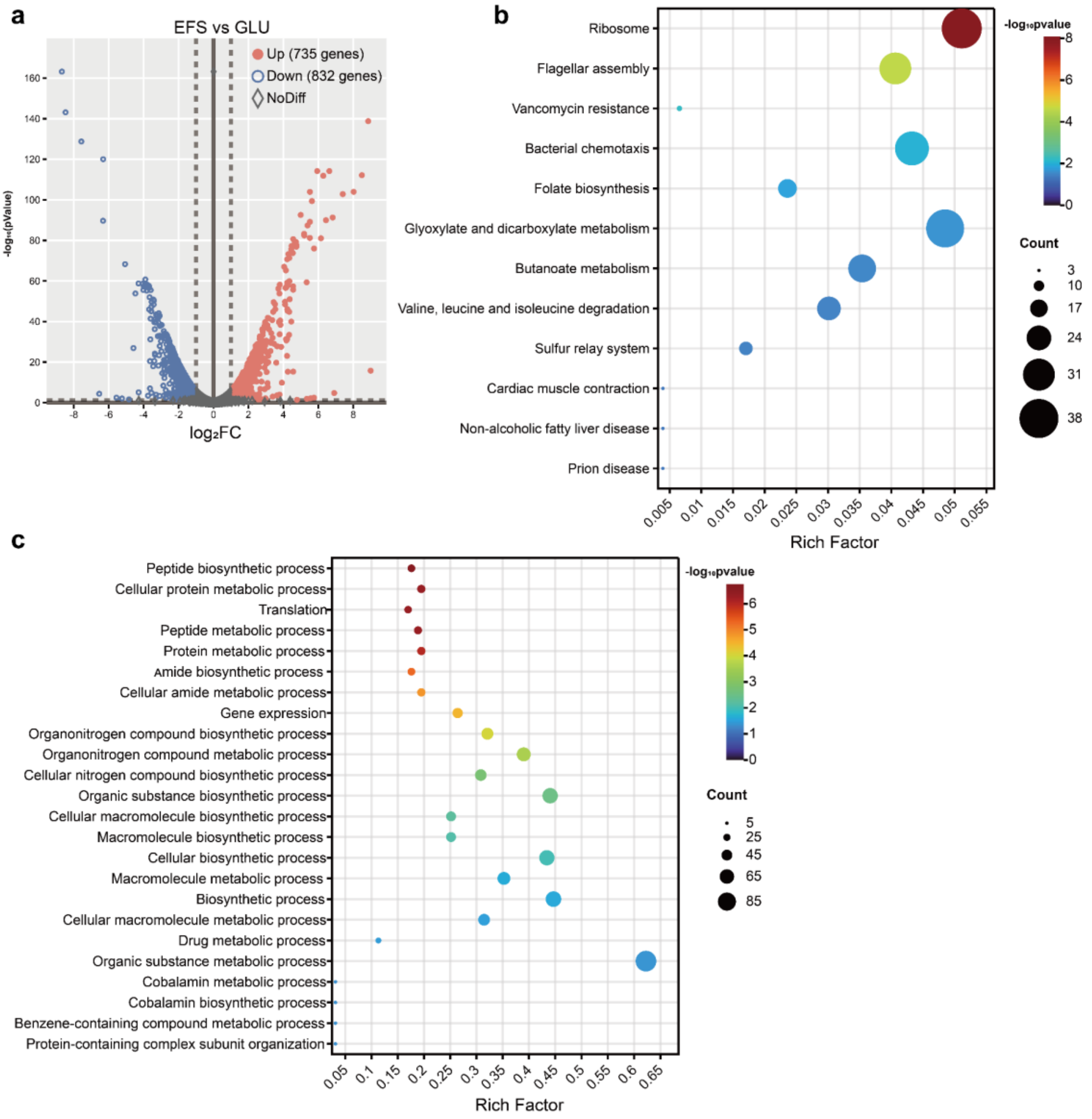

Supplementary Fig. 3: Differential transcriptome of NyZ480 by the induction of simple coumarin mixture. a Volcano plot of showing the differentially expressed genes (DEGs) in NyZ480 induced by simple coumarin mixtures (group EFS) compared with glucose-treated controls (group GLU). Significantly up- or down-regulated genes ($p < 0.05$, $|Log_2FC| > 1$) are shown as red and blue dots, respectively. **b** Kyoto Encyclopedia of Genes and Genomes (KEGG) enrichment analysis of all significant DEGs (both up- and down-regulated genes). **c** Enrichment analysis of all significant DEGs according to Gene Ontology (GO) terms associated with biological process.

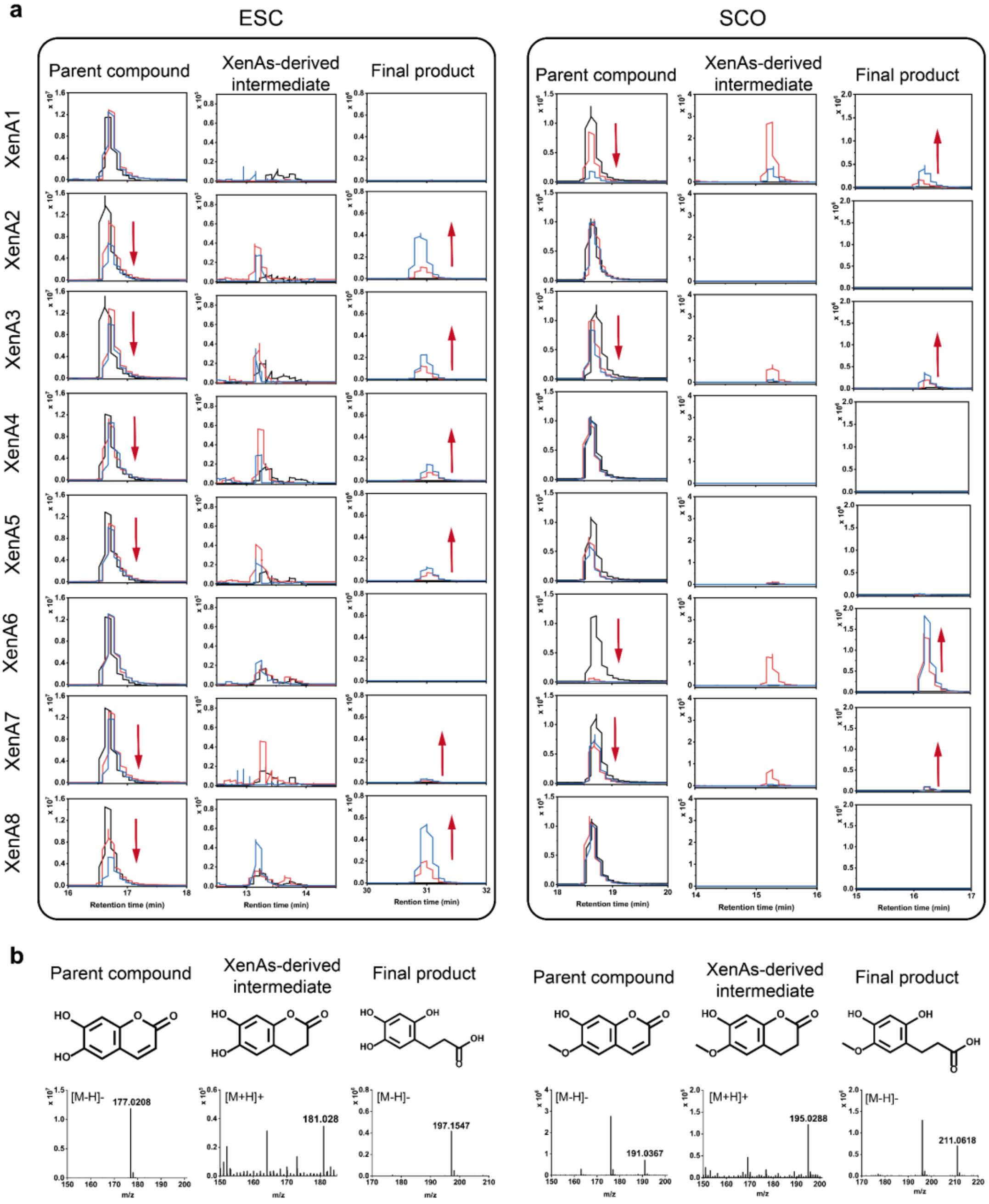

Supplementary Fig. 4: Simple coumarin degradation catalyzed by redundant XenA in NyZ480. **a** Time course of ESC and SCO biotransformation by *E. coli* cells containing each of the eight heterologously expressed XenA enzymes (XenA1-8). Reactions were monitored over two hours by extracted ion chromatograms (EICs) for the substrates (negative ion mode), the transient intermediates, and the final hydrolysis products. Red arrows indicate the decrease of ESC and SCO and the corresponding increase of the final products. **b** Mass spectrometric characterization of all chemicals involved. Shown are the authentic ESC and SCO standards (negative ion mode), the proposed transient intermediates generated by XenA catalysis (positive ion mode), and the final products resulting from spontaneous hydrolysis (negative ion mode).

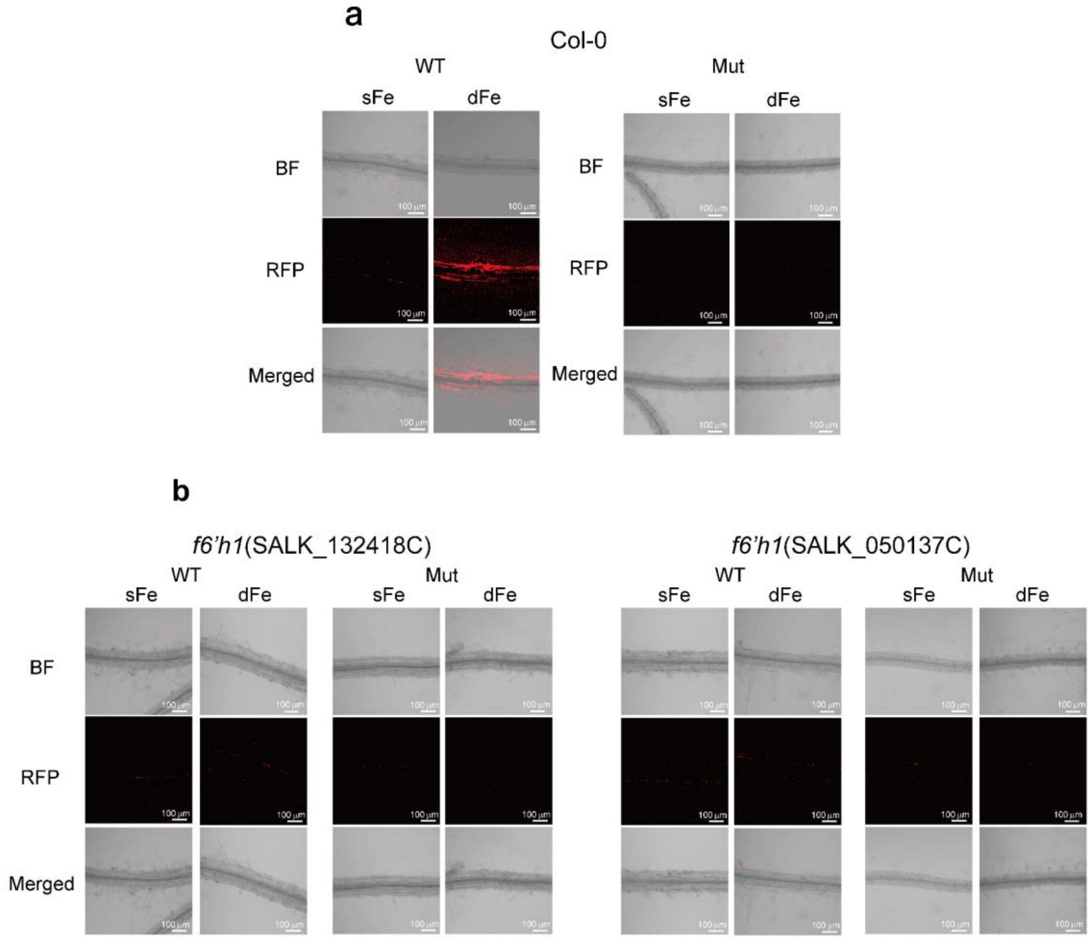

Supplementary Fig. 5: Confocal microscopic analysis of NyZ480 root colonization dynamics. Arabidopsis Col-0 (a) and *f6'h1* (b) seedlings were grown gnotobiotically for 14 days on iron-sufficient (sFe, 100 μM Fe^{2+}) or iron-deficient (dFe, 10 μM Fe^{3+}) ½ MS agar plates. Plant roots were inoculated with 20 μL of *rfp*-tagged wild-type (WT) or the mutant (Mut, namely NyZ480Δ8*xenA*sΔ*mhpB*) NyZ480 suspension (OD_{600}=0.003). Roots were sampled at 0 and 7 DPI, and imaged using confocal laser scanning microscopy (CLSM). Representative bright-field (BF) and red fluorescent protein (RFP) channel images are shown.

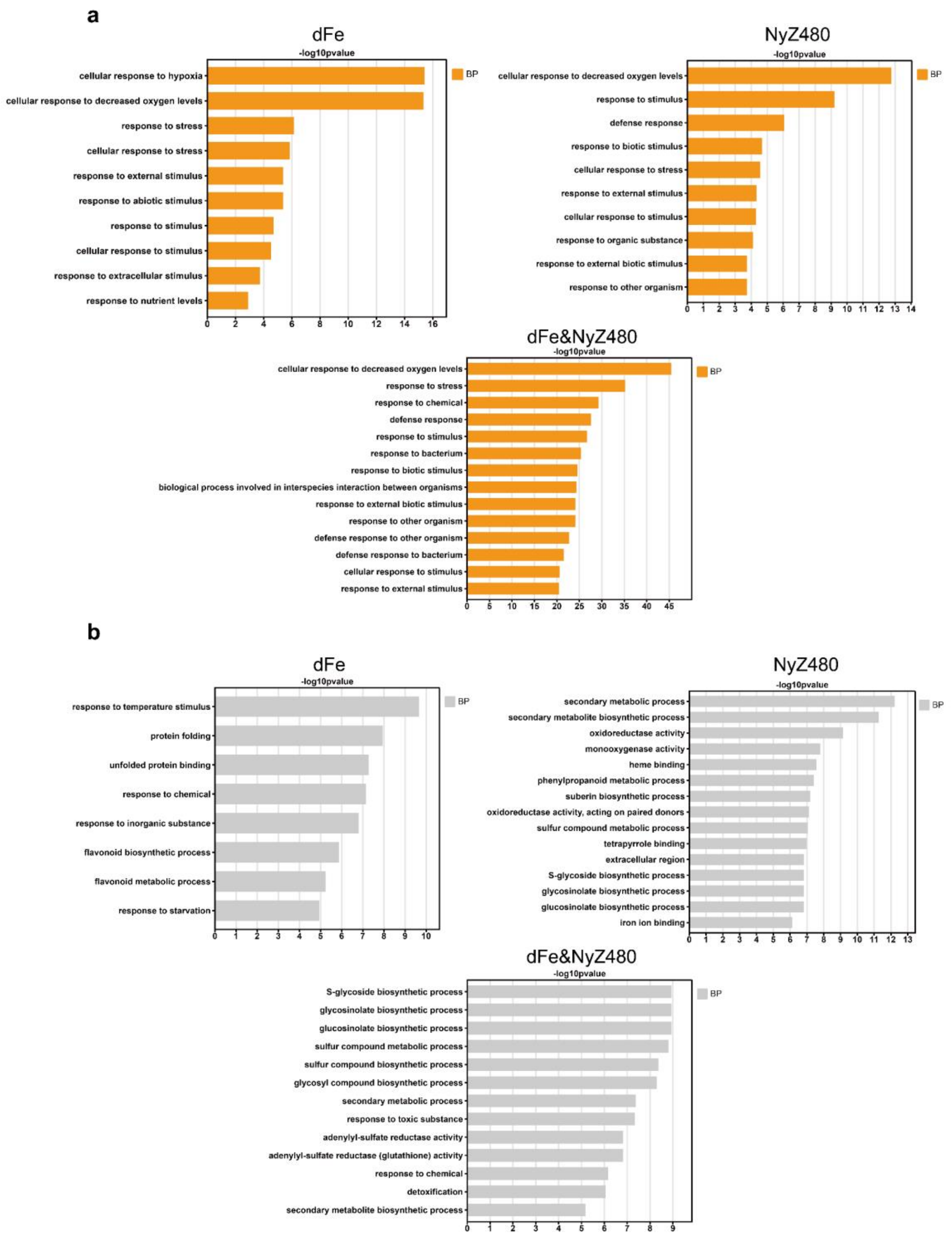

Supplementary Fig. 6: Gene Ontology (GO) enrichment analysis of differentially expressed genes (DEGs) in Arabidopsis Col-0 under different treatments. a Significantly enriched Biological Process (BP) terms among the up-regulated DEGs ($p < 0.05$, $log_2FC > 1$). **b** Significantly enriched Biological Process (BP) terms among the down-regulated DEGs ($p < 0.05$, $log_2FC < -1$).

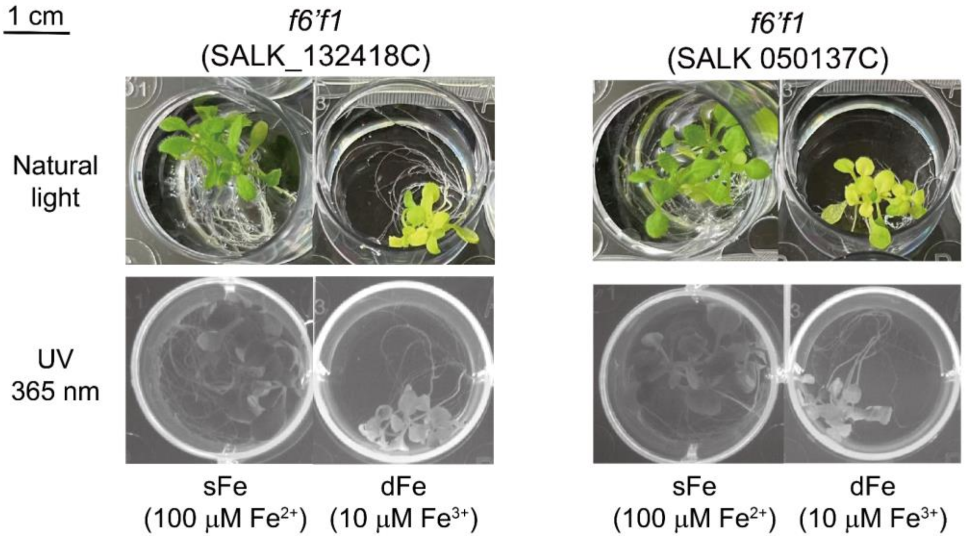

Supplementary Fig. 7: Phenotypic characterization of Arabidopsis mutant *f6'h1* with varying iron supplementation and preliminary determination of simple coumarin exudation by plants. Representative images of 18-day-old hydroponically grown plants under natural and UV (365 nm) light were taken to document phenotypic responses to sFe and dFe culture conditions, showing no emission of simple coumarins autofluorescence from *f6'h1*.

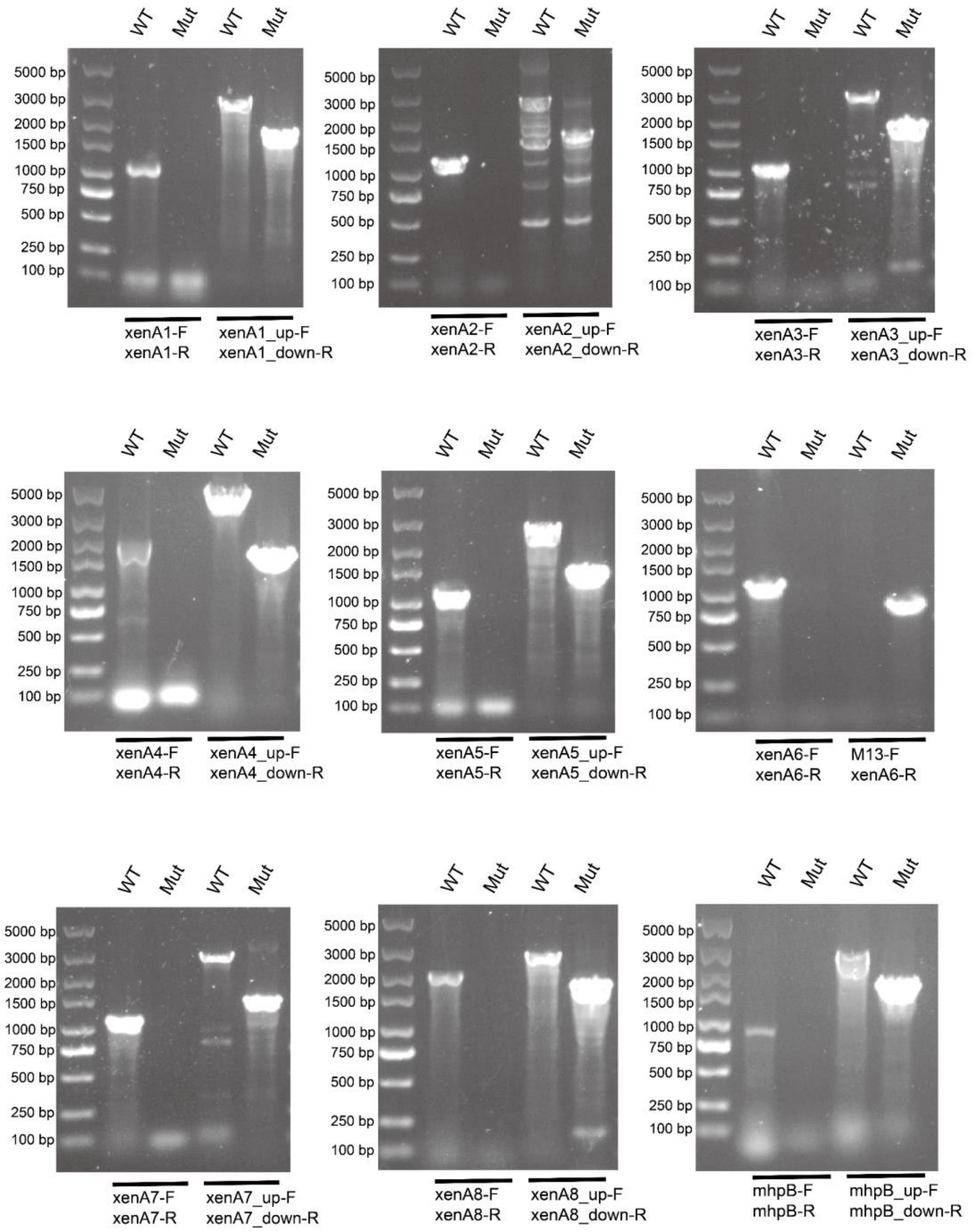

Supplementary Fig. 8: Verification of gene knockout in NyZ480 by PCR. The first lane in the gel electrophoresis images shows a DNA ladder. The second and third lanes show PCR products amplified from wild-type (WT) and mutant (Mut) genomic DNA, respectively, using primers specific to the target genes. For all genes except *xenA6*, the fourth and fifth lanes show PCR products amplified using primers flanking the target gene, confirming successful deletion. For *xenA6*, the fourth and fifth lanes display products amplified with primers M13 and xenA6-R, confirming insertion of the deletion vector pK18mobsacB into the *xenA6* locus.

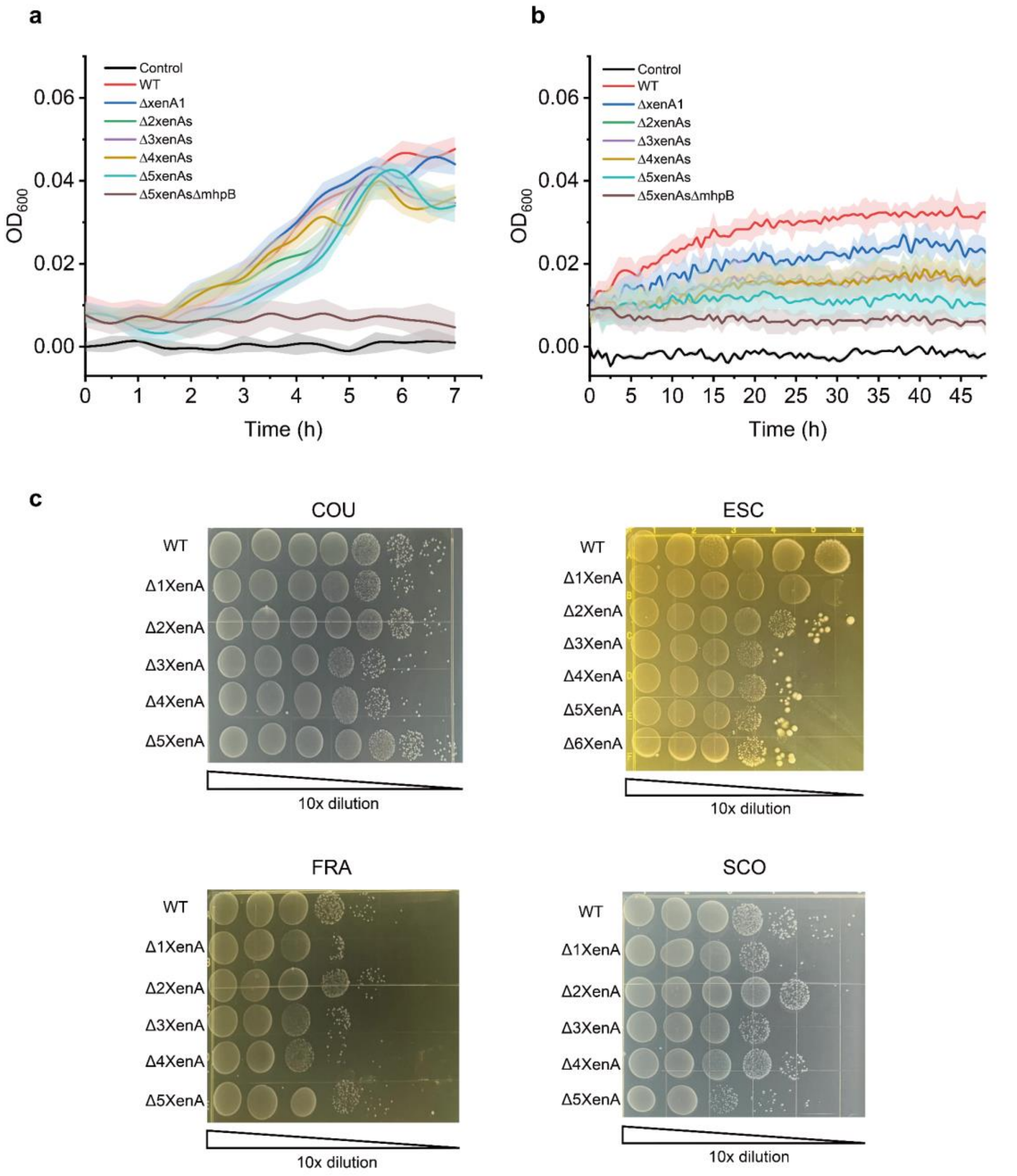

Supplementary Fig. 9: Simple coumarin utilization and resistance in the NyZ480 mutants. Bacterial growth of WT-NyZ480 and its mutants was measured with **a** COU or **b** FRA as the sole carbon source. Cultures were incubated at 30 °C with continuous medium shaking, and OD_{600} values were recorded every 30 min. Solid lines represent the mean values of three biological replicates (n=3), and shaded areas indicate the standard deviation (SD). Controls without bacterial inoculation were included. **c** Re-cultivation of WT-NyZ480 and its mutants on TSA plates containing 0.1 mM COU, ESC, FRA, or SCO, respectively. The abbreviations of NyZ480 mutants cultivated on TSA plates containing COU, FRA and SCO are as follows: ΔxenA1: NyZ480Δ*xenA1*, Δ2xenAs: NyZ480Δ*xenA1*Δ*xenA3*, Δ3xenAs: NyZ480Δ*xenA1*Δ*xenA3*Δ*xenA7*, Δ4xenAs: NyZ480Δ*xenA1*Δ*xenA3*Δ*xenA7*Δ*xenA5*, Δ5xenAs: NyZ480Δ*xenA1*Δ*xenA3*Δ*xenA7*Δ*xenA5*Δ*xenA6*, Δ6xenAsΔmhpB: NyZ480Δ*xenA1*Δ*xenA3*Δ *xenA7*Δ*xenA5*Δ*xenA6*Δ*mhpB*. For NyZ480 mutants cultivated on ESC plates, their abbreviations are as follows: ΔxenA1: NyZ480Δ*xenA8*, Δ2xenAs:

NyZ480Δ*xenA8*Δ*xenA2*, Δ3xenAs: NyZ480Δ*xenA8*Δ*xenA2*Δ*xenA3*, Δ4xenAs: NyZ480Δ*xenA8*Δ*xenA2*Δ*xenA3*Δ*xenA4*, Δ5xenAs: NyZ480Δ*xenA8*Δ*xenA2*Δ*xenA3*Δ*xenA4*Δ*xenA5*, Δ6xenAs: NyZ480Δ*xenA8*Δ*xenA2*Δ*xenA3*Δ*xenA4*Δ*xenA5*Δ*xenA7*.

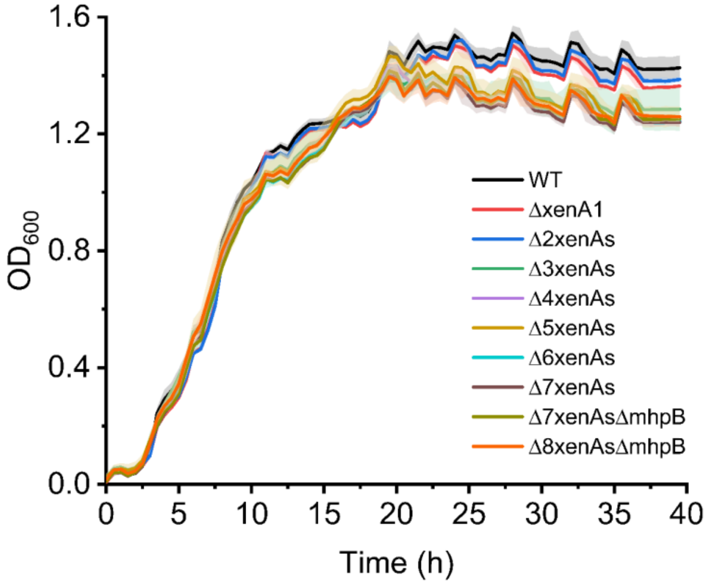

Supplementary Fig. 10: Growth of WT- and Mut-NyZ480 strains in LB medium. Single colonies of each strain were inoculated in 5 mL LB liquid medium and cultured overnight at 30 °C with shaking at 180 rpm. Bacterial cells were harvested (5,000 g, 5 min), and washed three times with PBS (pH 7.4). The pelleted cells were resuspended in 5 mL PBS buffer (pH 7.4), and used as seed cultures. Bacterial growth was monitored with the Bioscreen C system (Oy Growth Curves Ab Ltd., Helsinki, Finland). A volume of 300 µL of LB medium was dispensed into each well of a 96-well plate, followed by inoculation with 3 µL of the prepared seed culture solutions. Cultures were incubated at 30 °C with continuous medium shaking, and the OD_{600} was recorded every 30 min. Solid lines represent the mean values of three biological replicates (n=3), and shaded areas indicate the standard deviation (SD).

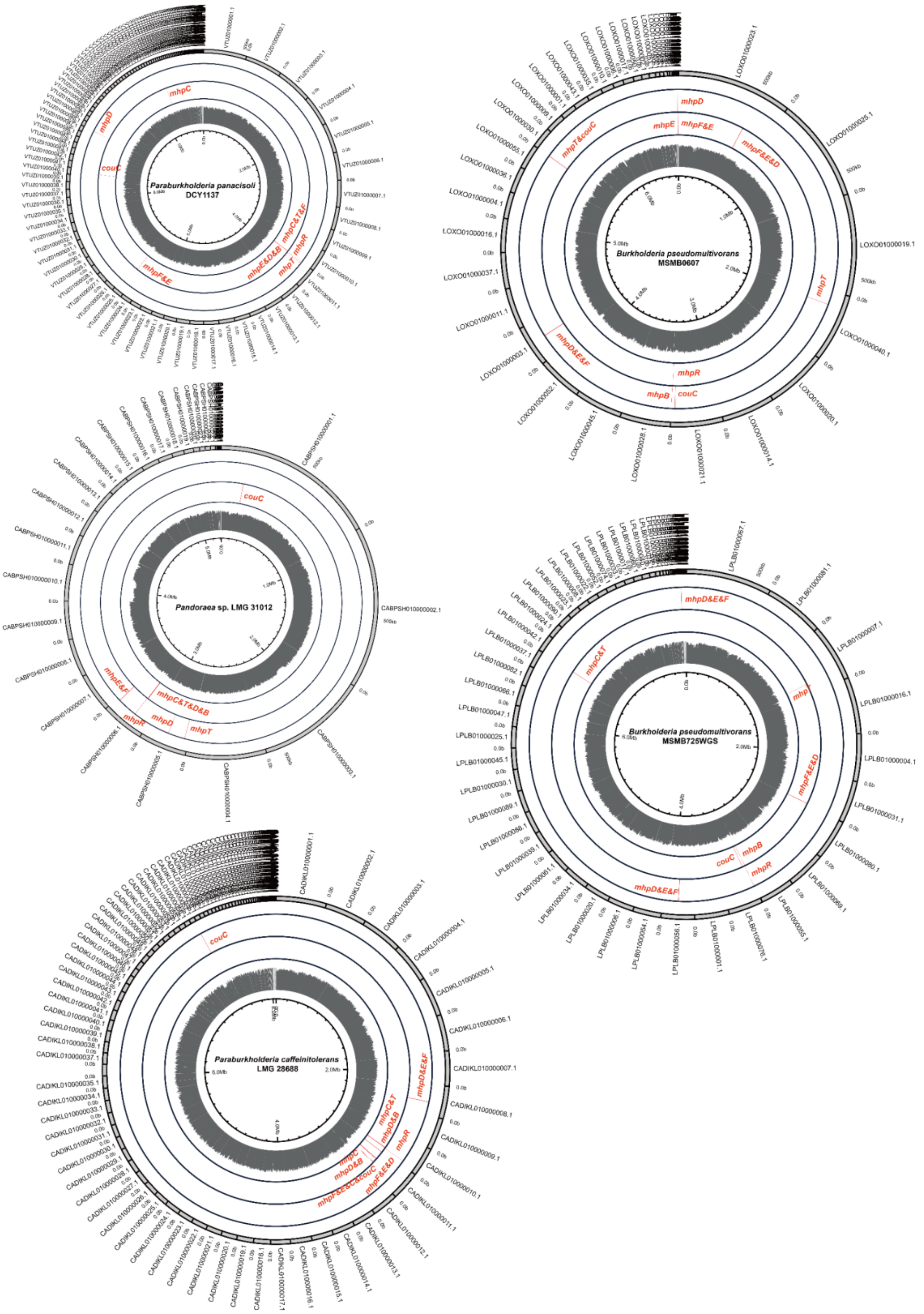

Supplementary Fig. 11: Scattered distribution of *couC* and *mph* cluster in five bacterial strains harboring the complete set of simple coumarin catabolic genes. Circular genome representations of *Burkholderia pseudomultivorans* MSMB0607, *Burkholderia pseudomultivorans* MSMB725WGS, *Pandoraea* sp. LMG 31012, *Paraburkholderia caffeinitolerans* LMG 28688, and *Paraburkholderia panacisoli* DCY113 are shown, and the catabolic genes are indicated in red.

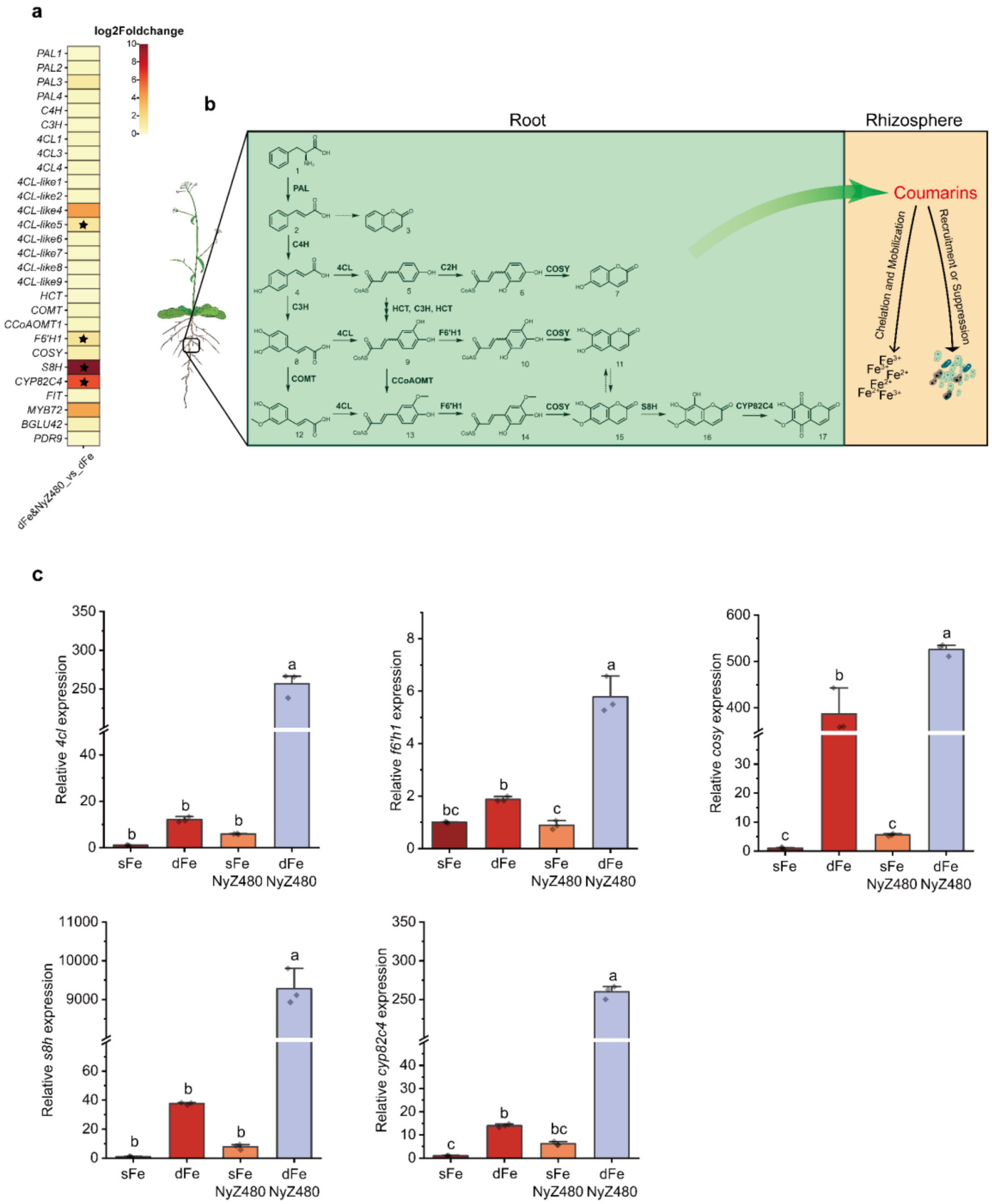

Supplementary Fig. 12: Transcriptional reprogramming of simple coumarin biosynthesis in Arabidopsis Col-0 under combinatorial treatment of iron deficiency and NyZ480 inoculation (dFe&NyZ480). a RNA-seq analysis showing $\log_2$FC in simple coumarin biosynthesis gene expression under combined treatment (dFe&NyZ480) relative to iron deficiency (dFe) alone. Asterisks indicate significant differences ($p < 0.05$). A two-tailed Wald test in DESeq2[1] was used to calculate p-values. **b** Schematic illustration of simple coumarin biosynthesis pathways in Arabidopsis. Numbers refer to the names of different compounds, 1: phenylalanine, 2: cinnamic acid, 3: coumarin, 4: *p*-coumaric acid, 5: *p*-coumaroyl-CoA, 6: 2-hydroxy-*p*-coumaroyl-CoA, 7: 7-hydroxy-coumarin (umbelliferone), 8: caffeic acid, 9: caffeoyl-CoA, 10: 6-hydroxy-caffeoyl-CoA, 11: esculetin, 12: ferrulate, 13: feruloyl-CoA, 14: 6-hydroxy-feruloyl-CoA, 15: scopoletin, 16:

fraxetin, 17: sideretin. Genes listed in **a** encode enzymes that catalyze each transformation in the simple coumarin biosynthetic pathways, which are abbreviated in **b** as follows: PAL, phenylalanine ammonia-lyase; C4H, cinnamate 4-hydroxylase; 4CL, 4-coumarate:CoA ligase; C2H, p-coumaroyl-CoA 2-hydroxylase; COSY, coumarin synthase; C3H, *p*-coumarate 3-hydroxylase; HCT, hydroxycinnamoyl-CoA shikimate/quinate hydroxycinnamoyl-transferase; F6′H1, feruloyl-CoA 6′-hydroxylase1; COMT, caffeic acid 3-O-methyltransferase; CCoAOMT, caffeoyl-CoA 3-O-methyltransferase; S8H, scopoletin 8-hydroxylase; CYP82C4, cytochrome P450 family B2 subfamily C polypeptide 4 enzyme. One dotted arrow indicates the uncharacterized enzyme. **c** RT-qPCR analysis of transcript levels of *4cl*, *f6′h1*, *cosy*, *s8h*, and *cyp82c4* in Arabidopsis Col-0 roots subjected to different treatments (sFe: iron sufficiency, dFe, sFe&NyZ480, and dFe&NyZ480). For each treatment, a pool of six seedlings constituted one biological replicate. Error bars indicate ± SD of three biological replicates (n=3). *AtTUB*^{2} was used as an internal control to normalize transcript levels. Statistical differences in panel **c** were determined by one-way ANOVA followed with Tukey post hoc test ($p < 0.05$). All exact p-values are provided in the Source Data file.

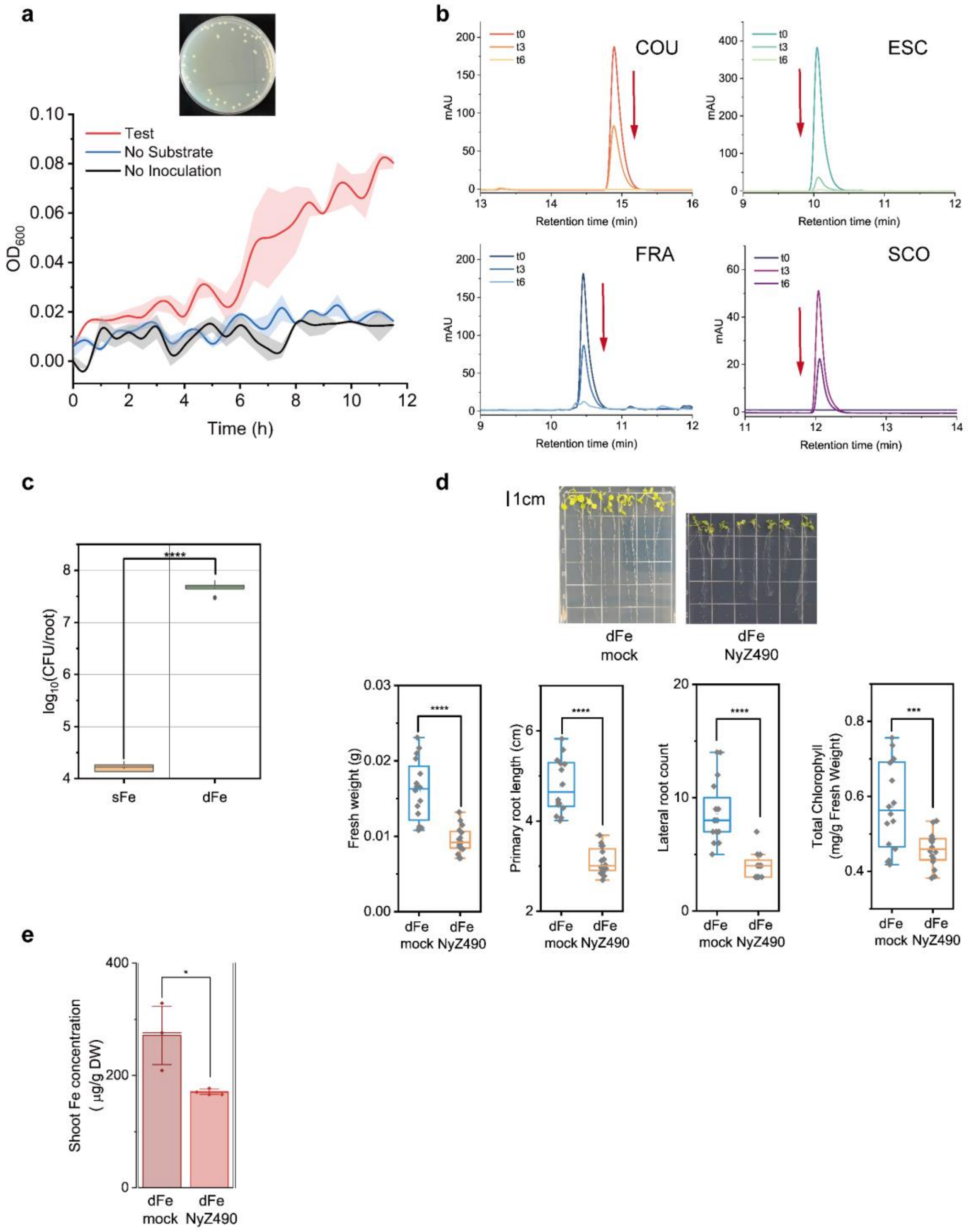

Supplementary Fig. 13: The Arabidopsis rhizosphere-derived coumarin degrader *Pseudomonas* sp. NyZ490 colonizes iron-deficient Arabidopsis Col-0 and compromises plant fitness. a Growth of NyZ490 with 1 mM COU as the sole carbon source. Control groups without COU (no substrate) or without NyZ490 (no inoculation) were included. **b** Degradation of COU, ESC, FRA, and SCO by NyZ490. HPLC chromatograms were captured during the degradation time course (0, 3, 6 h). Red arrows indicate the decline of substrates. **c** Log_{10}-transformed values of CFU of NyZ490 per root. Plants were gnotobiotically grown on sFe or dFe ½ MS agar plates for 14 days, 20 μL of NyZ490 suspension (OD_{600} = 0.003) was inoculated along the roots. CFU counts were

recorded at 7 DPI. Data represent three biological replicates (n=3), each with three technical replicates. **d** Phenotypic assessment of Arabidopsis Col-0 following NyZ490 inoculation under dFe conditions. 14-day-old plants were inoculated with 20 µL of NyZ490 suspension (OD_{600} = 0.003), or with 10 mM $MgCl_2$ as control (mock). Sampling was conducted at 7 DPI (n=16, each treatment comprised 16 seedlings). Fresh weight, primary root length, lateral root count and total chlorophyll were recorded. **e** Shoot iron concentration in iron-deficient (dFe) Col-0 plants treated with NyZ490 (NyZ490) or 10 mM $MgCl_2$ (mock). Shoot tissues were harvested at 7 DPI for iron concentration quantification by ICP-MS. For each treatment, a pool of three seedlings constituted one biological replicate. Error bars indicate ± SD of three biological replicates (n=3). Statistical differences in panel **c, d** and **e** were determined by two-tailed Student's t-test (*p < 0.05, ***p < 0.001, ****p < 0.0001). All exact p-values are provided in the Source Data file.

Supplementary tables

Supplementary Table 1: List of primers used for heterologous expression of XenA proteins

Primer name	Direction	Sequence (5'→3')	Target
XenA1-F	forward	CGCGCGGCAGCCATATGATGTCCGCGCTGTTCGA	*xenA1*
XenA1-R	reverse	GCTCGAATTCGGATCCTCAGCGATAGCGCTCGAGC	
XenA2-F	forward	CGCGCGGCAGCCATATGATGACTTCGGGTTATCCGCT	*xenA2*
XenA2-R	reverse	GCTCGAATTCGGATCCTTAGTCATATCGAATCGAATCGGGCC	
XenA3-F	forward	CGCGCGGCAGCCATATGATGACCACCCTTTTCGATCCGA	*xenA3*
XenA3-R	reverse	GCTCGAATTCGGATCCTTACATCCGCGGATAGTCGATGT	
XenA4-F	forward	CGCGCGGCAGCCATATGATGGGGAACCGTTTCATGGC	*xenA4*
XenA4-R	reverse	GCTCGAATTCGGATCCTCAAAGCTCCGCCGCCA	
XenA5-F	forward	CGCGCGGCAGCCATATGATGACCAGTAATCTGTTCAATCCTATTCGC	*xenA5*
XenA5-R	reverse	GCTCGAATTCGGATCCTTACGGGTGAGTCGGATAGTCGAT	
XenA6-F	forward	CGCGCGGCAGCCATATGATGAGCCTGCTGCTCGAG	*xenA6*
XenA6-R	reverse	GCTCGAATTCGGATCCCTAATCCCGCAAATCCGATTCGT	
XenA7-F	forward	CGCGCGGCAGCCATATGATGTCAACCAACCCGCTGT	*xenA7*
XenA7-R	reverse	GCTCGAATTCGGATCCTCAAGCCAGGGTCGACAATGC	
XenA8-F	forward	CGCGCGGCAGCCATATGATGGCATTCGAAGCAATGTTCC	*xenA8*
XenA8-R	reverse	GCTCGAATTCGGATCCTCAGAAGTCCTTGCACAGGCG	

Supplementary Table 2: Plasmids used in this study

Name	Description and characteristics	Source
pET-28a(+)	IPTG inducible expression vector; Km^r	Novagen
pET-xenA1	*xenA1* fragment inserted into pET-28a(+) between *NdeI* and *BamHI*	This study
pET-xenA2	*xenA2* fragment inserted into pET-28a(+) between *NdeI* and *BamHI*	This study
pET-xenA3	*xenA3* fragment inserted into pET-28a(+) between *NdeI* and *BamHI*	This study
pET-xenA4	*xenA4* fragment inserted into pET-28a(+) between *NdeI* and *BamH*	This study
pET-xenA5	*xenA5* fragment inserted into pET-28a(+) between *NdeI* and *BamHI*	This study
pET-xenA6	*xenA6* fragment inserted into pET-28a(+) between *NdeI* and *BamHI*	This study
pET-xenA7	*xenA7* fragment inserted into pET-28a(+) between *NdeI* and *BamHI*	This study
pET-xenA8	*xenA8* fragment inserted into pET-28a(+) between *NdeI* and *BamHI*	This study
pBBR1MCS-2	broad-host-range expression vector; mob^+, Km^r	[3]
pMCS-rfp	the red fluorescence protein-encoding gene fragment inserted into pBBR1MCS-2 between *KpnI* and *HindIII*	This study
pRK415	broad-host-range expression vector; $oriV_{RK2}$, $oriT_{RK2}$, Tc^R	[4]
pRK-rfp	the red fluorescence protein encoding gene fragment inserted into pRK415 between *EcoRI* and *HindIII*	This study
pK18mobsacB	gene deletion vector, mob^+, $sacB^+$, Km^r	[5]
pK-ΔxenA1	upstream and downstream fragments of *xenA1* fused and inserted into pK18mobsacB between *EcoRI* and *BamHI*	This study
pK-ΔxenA2	upstream and downstream fragments of *xenA2* fused and inserted into pK18mobsacB between *EcoRI* and *BamHI*	This study
pK-ΔxenA3	upstream and downstream fragments of *xenA3* fused and inserted into pK18mobsacB between *EcoRI* and *BamHI*	This study
pK-ΔxenA4	upstream and downstream fragments of *xenA4* fused and inserted into pK18mobsacB between *EcoRI* and *BamHI*	This study
pK-ΔxenA5	upstream and downstream fragments of *xenA5* fused and inserted into pK18mobsacB between *EcoRI* and *BamHI*	This study
pK-ΔxenA7	upstream and downstream fragments of *xenA7* fused and inserted into pK18mobsacB between *EcoRI* and	This study

	BamHI	
pK-ΔxenA8	upstream and downstream fragments of *xenA8* fused and inserted into pK18mobsacB between *EcoRI* and *BamHI*	This study
pK-ΔmhpB	upstream and downstream fragments of *mhpB* fused and inserted into pK18mobsacB between *EcoRI* and *BamHI*	This study
pK-ΔxenA6	the 500-bp gene fragment inserted into pK18mobsacB between *EcoRI* and *BamHI*	This study

Supplementary Table 3: List of primers used for construction of plasmids for gene knockout

Primer name	Direction	Sequence (5'→3')	Purpose
XenA1-up-F	forward	CCATGATTACGAATTGCAGATCCAGGGCGGC	Amplify upstream region of *xenA1*
XenA1-up-R	reverse	GTCTGTCCAAGCGGCCCTTTCGCG	
XenA1-down-F	forward	AGGGCCGCTTGGACAGACTCCAAGGGTTAACG	Amplify downstream region of *xenA1*
XenA1-down-R	reverse	CGACTCTAGAGGATCGGAGAAGGTCGCGACCC	
XenA2-up-F	forward	CGACTCTAGAGGATCGAGGTCGCGCGTCAGG	Amplify upstream region of *xenA2*
XenA2-up-R	reverse	TGGACTCTCCGCTCACACGCTCTGCCG	
XenA2-down-F	forward	CGTGTGAGCGGAGAGTCCAATGCAATTCGAGGA	Amplify downstream region of *xenA2*
XenA2-down-R	reverse	CCATGATTACGAATTCAACACCGCATCGGCG	
XenA3-up-F	forward	CGACTCTAGAGGATCAGCACCGGCCCGC	Amplify upstream region of *xenA3*
XenA3-up-R	reverse	GACCACGACTCGCAGCCCTGGTCGC	
XenA3-down-F	forward	GGGCTGCGAGTCGTGGTCCTTGTCAGAGC	Amplify downstream region of *xenA3*
XenA3-down-R	reverse	CCATGATTACGAATTTGATCGGCGTAGTCGCCC	
XenA4-up-F	forward	CGACTCTAGAGGATCTTTGAGACGTTTCTTAAATTGGCTTCTTTGTTTT	Amplify upstream region of *xenA4*
XenA4-up-R	reverse	AGCGAGTGGCGAAACGGTTCCCCATGTGGC	
XenA4-down-F	forward	AACCGTTTCGCCACTCGCTGTCCAAGT	Amplify downstream region of *xenA4*
XenA4-down-R	reverse	CCATGATTACGAATTGAGGGTAGCTCTGAGAGGCAC	
XenA5-up-F	forward	CGACTCTAGAGGATCCCGACGTTGCAGGCTTTG	Amplify upstream region of *xenA5*
XenA5-up-R	reverse	GAACTGAGTGCGGGCGTTGCTGGC	
XenA5-down-F	forward	CAACGCCCGCACTCAGTTCCTTTCAAGTCAGTGTTG	Amplify downstream region of *xenA5*
XenA5-down-R	reverse	CCATGATTACGAATTAGCCCTGTCGCGGG	
XenA7-up-F	forward	CGACTCTAGAGGATCGTACCCAGTGCCAGCCAG	Amplify upstream region of *xenA7*

XenA7-up-R	reverse	GCGCGCCCTCGTCAGCGCAGGGGC	
XenA7-down-F	forward	CTGACGAGGGCGCGCGTCC	Amplify downstream region of *xenA7*
XenA7-downR	reverse	CCATGATTACGAATTTAGTGGGCGGGTTTCGC	
XenA8-up-F	forward	CGACTCTAGAGGATCAAGGCGTGCTTCATCGGG	Amplify upstream region of *xenA8*
XenA8-up-R	reverse	GAGAGCGTCTCGCTCCGCCTCTCTAGAGG	
XenA8-down-F	forward	GCGGAGCGAGACGCTCTCTCCGACTTACAACG	Amplify downstream region of *xenA8*
XenA8-down-R	reverse	CCATGATTACGAATTTCGGCATTCGCACTGTCG	
MhpB-up-F	forward	CGACTCTAGAGGATCTGAATCTCGCCGAGGCGAT	Amplify upstream region of *mhpB*
MhpB-up-R	reverse	GGAAAGCGCTCCACACCTTCACGGAGATTCAATCG	
MhpB-down-F	forward	TGAAGGTGTGGAGCGCTTTCCTCAATGCCT	Amplify downstream region of *mhpB*
MhpB-down-R	reverse	CCATGATTACGAATTTGGCGATTTTGCCATTGCG	
XenA6-del-F	forward	CGACTCTAGAGGATCCCCACGCTGGGCGC	Amplify a 500-bp fragment within *xenA6*
XenA6-del-R	reverse	CCATGATTACGAATTGCCACCGGAGGAAACATCG	

Supplementary Table 4: List of primers used for verification of gene knockout of NyZ480

Primer name	Direction	Sequence (5'→3')	Purpose
XenA1-1st-F	forward	CGCCGGCACTCAAGCACGACAAT	Verify the first round of gene knockout of *xenA1*
XenA1-1st-R	reverse	TCGCCATTCAGGCTGCGCAACTG	
XenA1-2nd-F	forward	CAGGCGCTCGATGCCCTGCA	Verify the second round of gene knockout of *xenA1*
XenA1-2nd-R	reverse	TTGCCTCGCAAGACCGCACCAT	
XenA2-1st-F	forward	TGCATGTTCTGCCCGGT	Verify the first round of gene knockout of *xenA2*
XenA2-1st-R	reverse	ATGCTTCCGGCTCGTATGT	
XenA2-2nd-F	forward	TGCATGTTCTGCCCGGT	Verify the second round of gene knockout of *xenA2*
XenA2-2nd-R	reverse	AGCGCATGGCCGATGC	
XenA3-1st-F	forward	CGTAACGATTCCCACGACGAC	Verify the first round of gene knockout of *xenA3*
XenA3-1st-R	reverse	CTCACTCATTAGGCACCCCAGG	
XenA3-2nd-F	forward	CCGGCACCCCTGATGATGAC	Verify the second round of gene knockout of *xenA3*
XenA3-2nd-R	reverse	CACGCGATAGAAGTCCTGCG	
XenA4-1st-F	forward	GCCATGGCTGGGAGCAAGAG	Verify the first round of gene knockout of *xenA4*
XenA4-1st-R	reverse	TGGCACGACAGGTTTCCCGA	
XenA4-2nd-F	forward	GCCATGGCTGGGAGCAAGAG	Verify the second round of gene knockout of *xenA4*
XenA4-2nd-R	reverse	TGGTGGCGCAGCTTGAACC	
XenA5-1st-F	forward	CCAATGCGGTGGCCATCAG	Verify the first round of gene knockout of *xenA5*
XenA5-1st-R	reverse	TGTGTGGAATTGTGAGCGGATAA	
XenA5-2nd-F	forward	GGCCGAACAAGACCAGCAC	Verify the second round of gene knockout of *xenA5*
XenA5-2nd-R	reverse	CCTGGAAAGCGGCCTCT	
XenA7-1st-F	forward	GGGTTTTCCCAGTCACGACGT	Verify the first round of gene knockout of *xenA7*

XenA7-1st-R	reverse	CGCTAAGCTCAGAAAGTCGCCA	
XenA7-2nd-F	forward	ACCGCCGCCACACTG	Verify the second round of gene knockout of *xenA7*
XenA7-2nd-R	reverse	GCTTGAACGAGCGCCTTT	
XenA8-1st-F	forward	TGCACGCAGGCATCGAAGC	Verify the first round of gene knockout of *xenA8*
XenA8-1st-R	reverse	ATTAATGCAGCTGGCACGACA	
XenA8-2nd-F	forward	TGCACGCAGGCATCGAAGC	Verify the second round of gene knockout of *xenA8*
XenA8-2nd-R	reverse	CCGAGGCCATCGAGTACAC	
MhpB-1st-F	forward	CCTCTTCGCTATTACGCCAGC	Verify the first round of gene knockout of *mhpB*
MhpB-1st-R	reverse	TGGCCACACTGTGAATATGGC	
MhpB-2nd-F	forward	CAAGCGCTCAAGGGTCTCTTG	Verify the second round of gene knockout of *mhpB*
MhpB-2nd-R	reverse	TGGCCACACTGTGAATATGGC	
XenA6-delv-F	forward	GTAAAACGACGGCCAGT	Verify the inactivation of *xenA6*
XenA6-delv-R	reverse	GCTCAGCGAAGCGCGT	

Supplementary Table 5: Acquisition parameters of Agilent 6460 Triple Quad MS system for quantification of simple coumarins

**Compound**	**Precursor ion**	**Product ion**	**Dwell**	**Fragmentor voltage**	**Collision energy**	**Cell accelerator voltage**	**Polarity**
Coumarin	147	102.9	200	135	10	5	Positive
Esculetin	177	133	200	135	15	5	Negative
Fraxetin	207	191.9	200	135	15	5	Negative
Scopoletin	191	175.9	200	135	12	5	Negative

References

1. Love, M. I., Huber, W. & Anders, S. Moderated estimation of fold change and dispersion for RNA-seq data with DESeq2. *Genome Biol.* **15**, 550 (2014).
2. Lian, H. *et al.* Redundant and specific roles of individual MIR172 genes in plant development. *PLoS Biol* **19**, e3001044 (2021).
3. Kovach, M. E. *et al.* Four new derivatives of the broad-host-range cloning vector pBBR1MCS, carrying different antibiotic-resistance cassettes. *Gene* **166**, 175–176 (1995).
4. Keen, N. T., Tamaki, S., Kobayashi, D. & Troilinger, D. Improved brood-host-range plasmids for DNA cloning in Gram-negative bacteria. *GENE* **70**, 191–197 (1988).
5. Schgfer, A. *et al.* Small mobilizable multi-purpose cloning vectors derived from the *Escherichia coli* plasmids pK18 and pK19: selection of defined deletions in the chromosome of *Corynebacterium glutumicum*. *GENE* **145**, 69–73 (1994).
