# Supplementary material for: Rhizobacteria opportunistically boost colonization and impair plant fitness by degrading plant-derived coumarins under iron deficiency
Source: Nat Commun. 2026 Mar 25;17:4398. doi: 10.1038/s41467-026-71037-3 (PMC13181038; doi:10.1038/s41467-026-71037-3)
Supplement: Supplementary file 2 — Description of Additional Supplementary Information [file 41467_2026_71037_MOESM2_ESM.pdf]

Description of Additional Supplementary Files

File Name: Supplementary Data 1
Description: Quantification of simple coumarins exuded by *f6'h1* under iron deficiency.

File Name: Supplementary Data 2
Description: Occurrence of *xenA* homologs in bacterial genomes retrieved from IMG/M database.

File Name: Supplementary Data 3
Description: Occurrence of coumarin catabolic genes in bacterial genomes across different ecological niches.

File Name: Supplementary Data 4
Description: Genomic locations of *xenA* homologs in 23 strains harboring complete coumarin catabolic genes.

File Name: Supplementary Data 5
Description: Genomic locations of *couC* and *mhp* genes in 23 strains harboring complete coumarin catabolic genes.
