# Supplementary material for: Rhizobacteria opportunistically boost colonization and impair plant fitness by degrading plant-derived coumarins under iron deficiency
Source: Nat Commun. 2026 Mar 25;17:4398. doi: 10.1038/s41467-026-71037-3 (PMC13181038; doi:10.1038/s41467-026-71037-3)
Supplement: Supplementary file 8 — Reporting Summary [file 41467_2026_71037_MOESM8_ESM.pdf]

nature portfolio

Corresponding author(s): Ning-Yi Zhou

Last updated by author(s): 2026-03-08

Reporting Summary

Nature Portfolio wishes to improve the reproducibility of the work that we publish. This form provides structure for consistency and transparency in reporting. For further information on Nature Portfolio policies, see our Editorial Policies and the Editorial Policy Checklist.

Statistics

For all statistical analyses, confirm that the following items are present in the figure legend, table legend, main text, or Methods section.

n/a	Confirmed	
☐	☒	The exact sample size (*n*) for each experimental group/condition, given as a discrete number and unit of measurement
☐	☒	A statement on whether measurements were taken from distinct samples or whether the same sample was measured repeatedly
☐	☒	The statistical test(s) used AND whether they are one- or two-sided<br>*Only common tests should be described solely by name; describe more complex techniques in the Methods section.*
☒	☐	A description of all covariates tested
☐	☒	A description of any assumptions or corrections, such as tests of normality and adjustment for multiple comparisons
☐	☒	A full description of the statistical parameters including central tendency (e.g. means) or other basic estimates (e.g. regression coefficient) AND variation (e.g. standard deviation) or associated estimates of uncertainty (e.g. confidence intervals)
☐	☒	For null hypothesis testing, the test statistic (e.g. *F*, *t*, *r*) with confidence intervals, effect sizes, degrees of freedom and *P* value noted<br>*Give P values as exact values whenever suitable.*
☒	☐	For Bayesian analysis, information on the choice of priors and Markov chain Monte Carlo settings
☒	☐	For hierarchical and complex designs, identification of the appropriate level for tests and full reporting of outcomes
☒	☐	Estimates of effect sizes (e.g. Cohen's *d*, Pearson's *r*), indicating how they were calculated

Our web collection on statistics for biologists contains articles on many of the points above.

Software and code

Policy information about availability of computer code

Data collection	All annotated bacterial genomes were retrieved from the IMG/M database (https://img.jgi.doe.gov/).
Data analysis	Bacterial genomes were downloaded using NCBI Datasets (https://github.com/ncbi/datasets); Amino acid sequences of eight XenAs were aligned by MUSCLE (v5.1) and used to construct a profile Hidden Markov Model (pHMM) with the hmmbuild program of HMMER (v3.4), the obtained pHMM was used to inspect potential XenA in bacterial genomes using hmmsearch program of HMMER (v3.4); Amino acid sequences of the downstream catabolic enzymes CouC and MhpBCDEFRT were searched in bacterial genomes using Diamond (v2.1.13); Organization of coumarin catabolic genes was analyzed by Cblaster (v1.3.18); Phylogenetic analysis was performed with GTDB-Tk (v2.4.0).

For manuscripts utilizing custom algorithms or software that are central to the research but not yet described in published literature, software must be made available to editors and reviewers. We strongly encourage code deposition in a community repository (e.g. GitHub). See the Nature Portfolio guidelines for submitting code & software for further information.

Data

Policy information about availability of data

All manuscripts must include a data availability statement. This statement should provide the following information, where applicable:

- Accession codes, unique identifiers, or web links for publicly available datasets
- A description of any restrictions on data availability
- For clinical datasets or third party data, please ensure that the statement adheres to our policy

Transcriptome data of NyZ480 and Arabidopsis plants have been deposited at NCBI under the BioProject accession PRJNA951349 and PRJNA1204056, respectively.

Source Data are provided with this paper.

Research involving human participants, their data, or biological material

Policy information about studies with human participants or human data. See also policy information about sex, gender (identity/presentation), and sexual orientation and race, ethnicity and racism.

Reporting on sex and gender	not applicable
Reporting on race, ethnicity, or other socially relevant groupings	not applicable
Population characteristics	not applicable
Recruitment	not applicable
Ethics oversight	not applicable

Note that full information on the approval of the study protocol must also be provided in the manuscript.

Field-specific reporting

Please select the one below that is the best fit for your research. If you are not sure, read the appropriate sections before making your selection.

☐ Life sciences ☐ Behavioural & social sciences ☒ Ecological, evolutionary & environmental sciences

For a reference copy of the document with all sections, see nature.com/documents/nr-reporting-summary-flat.pdf

Ecological, evolutionary & environmental sciences study design

All studies must disclose on these points even when the disclosure is negative.

Study description	This study demonstrates that a rhizosphere-isolated strain of Pseudomonas opportunistically boosts enhanced colonization on iron-deficient Arabidopsis plants by utilizing and detoxifying plant-derived simple coumarins, and consequently impairs plant iron acquisition and overall fitness.
Research sample	We tested Pseudomonas sp. strain NyZ480 for its capacity of degrading and utilizing simple coumarins, and its colonization on Arabidopsis Col-0 and f6'h1 (SALK_132418C, SALK_050137C). This was done through assays of bacterial growth monitoring, biodegradation, RNA-sequencing analysis, imaging using confocal laser scanning microscopy, etc. We heterogeneously expressed the redundant XenAs in NyZ480, and performed biotransformation against simple coumarin compounds. We generated mutants of Pseudomonas sp. strain NyZ480, and also tested their capacity of degrading and utilizing simple coumarins, and their colonization on Arabidopsis Col-0 and f6'h1 (SALK_132418C, SALK_050137C). We surveyed the occurrence and abundance of the coumarin-degradation gene xenA in environmental bacterial genomes.
Sampling strategy	This study did not involve direct sampling of environmental samples.
Data collection	Data from biodegradation and biotransformation assays were recorded using LC-MS (Agilent 1290 Infinity II / 6545 QTOF); Quantification of simple coumarins secreted by Arabidopsis was conducted using LC-MS (Agilent 1260 / 6470 QQQ); Bacterial genomes were downloaded from the IMG/M database (https://img.jgi.doe.gov/); Bacterial growth data were recorded using Bioscreen C system (Oy Growth Curves Ab Ltd., Helsinki, Finland); Plant phenotypic data were recorded using images; Raw RNA sequencing data were generated with the Illumina NovaSeq PE150 platform; RT-qPCR data were obtained using CFX96 real-time PCR detection system (Bio-Rad); Arabidopsis iron concentrations were measured by ICP-MS (NexION2000G).
Timing and spatial scale	Timing is dependent on different experiments: bacterial growth of NyZ480 using simple coumarins was monitored with 48 hours; NyZ480 degradation of simple coumarins were conducted in 6 hours; biotransformation assays of XenAs were recorded within 2 hours; NyZ480 colonization on Arabidopsis was monitored in 12 days.<br>All experiments were conducted on a laboratory scale at Shanghai Jiao Tong University between 2022 and 2025.
Data exclusions	No data were excluded from the analyses.
Reproducibility	All biological experiments were repeated independently to ensure reproducibility in this study .
Randomization	No Randomization was required in this study. All experiments were performed under controlled laboratory conditions. Data points were not assigned to experimental groups randomly.
Blinding	Blinding was not applicable to this study. All experiments and analyses were standardized and not influenced by investigators' expectations.

Did the study involve field work? ☐ Yes ☒ No

Reporting for specific materials, systems and methods

We require information from authors about some types of materials, experimental systems and methods used in many studies. Here, indicate whether each material, system or method listed is relevant to your study. If you are not sure if a list item applies to your research, read the appropriate section before selecting a response.

Materials & experimental systems

n/a	Involved in the study
☒	☐ Antibodies
☒	☐ Eukaryotic cell lines
☒	☐ Palaeontology and archaeology
☒	☐ Animals and other organisms
☒	☐ Clinical data
☒	☐ Dual use research of concern
☐	☒ Plants

Methods

n/a	Involved in the study
☒	☐ ChIP-seq
☒	☐ Flow cytometry
☒	☐ MRI-based neuroimaging

Dual use research of concern

Policy information about dual use research of concern

Hazards

Could the accidental, deliberate or reckless misuse of agents or technologies generated in the work, or the application of information presented in the manuscript, pose a threat to:

No	Yes
☒	☐ Public health
☒	☐ National security
☒	☐ Crops and/or livestock
☒	☐ Ecosystems
☒	☐ Any other significant area

Experiments of concern

Does the work involve any of these experiments of concern:

No	Yes
☒	☐ Demonstrate how to render a vaccine ineffective
☒	☐ Confer resistance to therapeutically useful antibiotics or antiviral agents
☒	☐ Enhance the virulence of a pathogen or render a nonpathogen virulent
☒	☐ Increase transmissibility of a pathogen
☒	☐ Alter the host range of a pathogen
☒	☐ Enable evasion of diagnostic/detection modalities
☒	☐ Enable the weaponization of a biological agent or toxin
☒	☐ Any other potentially harmful combination of experiments and agents

Plants

Seed stocks	Arabidopsis Col-0 and f6’h1 (SALK_132418C, SALK_050137C) were used in this study.
Novel plant genotypes	No novel plant genotypes were generated in this study.
Authentication	Not applicable
